# Impact of a robotic system on intra‐abdominal infectious complications after minimally invasive gastrectomy in patients with gastric cancer: A propensity score matching analysis regarding visceral obesity

**DOI:** 10.1002/ags3.12748

**Published:** 2023-10-11

**Authors:** Naoshi Kubo, Katsunobu Sakurai, Tsuyoshi Hasegawa, Yutaka Tamamori, Yasuhito Iseki, Takafumi Nishii, Sadatoshi Shimizu, Toru Inue, Yukio Nishiguchi, Kiyoshi Maeda

**Affiliations:** ^1^ Department of Gastroenterological Surgery Osaka City General Hospital Osaka Japan; ^2^ Department of Gastroenterological Surgery Osaka Metropolitan University Graduate School of Medicine Osaka Japan

**Keywords:** gastric cancer, robotic gastrectomy, visceral obesity

## Abstract

**Background:**

The efficacy of robotic gastrectomy (RG) on postoperative complications in patients with gastric cancer (GC) is unclear in terms of the volume of intra‐abdominal fat tissue.

**Patients and Methods:**

We enrolled 403 patients with GC who had minimally invasive surgery between January 2015 and July 2022. During this time, 197 RG and 206 laparoscopic gastrectomies (LG) were performed on the study participants. According to the computed tomography scan, patients were defined as having or not having visceral obesity based on the visceral fatty area (VFA). In each high and low VFA group, we compared short‐term outcomes between the RG group and LG group.

**Results:**

After PS matching for patients with high VFA, the two groups were well matched, with 71 cases in both the RG and LG groups. The median surgical time in the RG group was significantly longer (420 vs. 365 min, *p* < 0.001). However, the RG group had a significantly lower rate of severe intra‐abdominal infectious complications (IAIC), such as anastomotic leakage, pancreatic fistula, and intra‐abdominal abscess (1.4% vs. 15.4%, *p* = 0.004). However, among the 77 patients with low VFA values, we found no significant difference in the rate of severe IAIC between the two groups (1.1% in the RG group vs. 2.6% in the LG group, *p* = 1.00).

**Conclusion:**

RG may be a viable alternative to LG because of the lower postoperative IAIC for patients with visceral obesity and GC. However, RG may not benefit non‐obese patients.

## Graphical abstract

The robotic system reduced intra‐abdominal infectious complications after gastrectomy for viscerally obese GC patients. RG could be an alternative to LG in patients with visceral obesity and GC; however, RG may not benefit non‐viscerally obese patients.

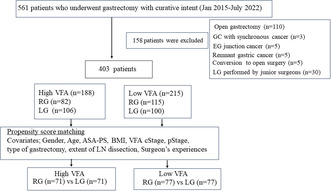

## 1 INTRODUCTION

Gastric cancer (GC) ranks third and fifth in terms of the annual number of cancer deaths and cancer incidence worldwide.^1^ The cornerstone of treatment for localized GC is surgical resection of the primary tumor and regional lymph nodes. Recently, two types of minimally invasive surgical approaches, including laparoscopic gastrectomy (LG) and robotic gastrectomy (RG), are used to complete GC resection.

Some surgeons^2^, ^3^ report that the robotic system has many advantages over laparoscopic surgery, such as articulated instruments, magnified clear view via a 3D camera, and tremor filtering. These advantages enable complete endoscopic resection of GC without increasing surgeon burden.^4^ However, the clear clinical benefits of RG compared to LG are unclear. Some surgeons espouse the merits of RG—including less blood loss, more retrieved lymph nodes, fewer intra‐abdominal complications, and shorter hospital stays compared with LG.^5^, ^6^, ^7^, ^8^ Other surgeon's feel that RG has no significant short‐ or long‐term advantages over LG.^9^, ^10^ RG may be helpful during more technically demanding cases such as surgery in patients with obesity or more‐advanced disease. However, there is little evidence to support the clinical advantages of RG for those technically demanding cases.

Intra‐abdominal excess fatty tissues tear easily and result in easy bleeding during surgery. Excess fat around the pancreas makes it difficult to recognize the pancreatic parenchyma border. There are some reports in which viscerally obese patients are closely associated with more bleeding, longer operation time, and more postoperative complications, especially pancreas‐related complications.^11^, ^12^

Recently the number of obese patients has increased globally,^13^, ^14^ as has the number of patients with visceral obesity and GC requiring surgery. We hypothesized that RG has clinical benefits over LG for patients with obesity because of the robot's mechanical advantage. We compared short‐term outcomes between RG and LG for patients with and without visceral obesity.

## 2 PATIENTS AND METHODS

### 2.1 Patients

**FIGURE 1:**
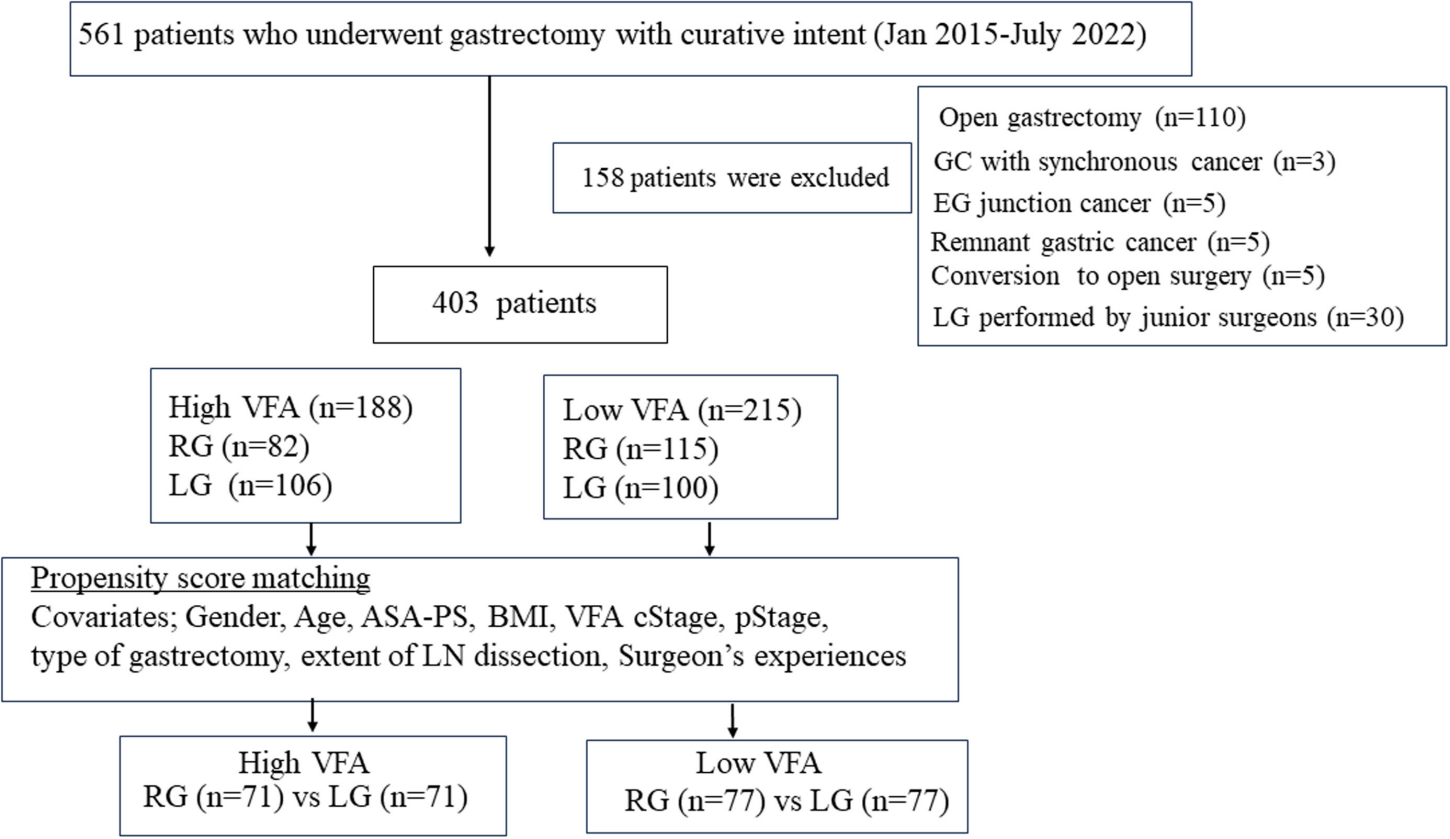
Study flow and patient selection. Between January 2015 and July 2022, 561 patients with GC underwent gastrectomy at Osaka City General Hospital. We excluded 158 patients with GC for various reasons (110 underwent open surgery, five had remnant GC, five were converted from RG or LG to open surgery, three had GC plus another cancer, five had esophageal junction cancer, and 30 patients whose surgeries were performed by junior surgeons). Finally, 197 RG and 206 LG cases were eligible for this study.

Figure 1 shows the flow of patient selection in this study. Between January 2015 and July 2022, 561 patients with GC underwent curative surgical resection at Osaka City General Hospital. We excluded 110 patients with a suspected T4 tumor or bulky metastatic LNs who underwent open surgery, five with esophagogastric junction cancer, including Siewert Type I and Type II, five with remnant GC, and three with GC with another synchronous cancer. Open surgery was indicated for patients with suspected T4 tumors or bulky metastatic LNs. Minimally invasive surgery (MIS), such as LG or RG, is used with patients without highly advanced GC, such as those with T4 tumors and bulky metastatic LNs. Five patients (three RG cases and two LG cases) who were converted to open surgery was excluded from this study. In this series, three of the 197 patients (1.5%) in the RG group and two of the 206 patients (0.9%) in the LG group were converted to open surgery, with no significant difference in the rate between the two groups (*p* = 0.78). Of the three patients in the RG group, two cases were converted to open surgery because of uncontrollable intra‐abdominal bleeding and the other because of intra‐abdominal adhesion. In the LG group, uncontrollable bleeding and intra‐abdominal adhesion led to conversion to open surgery in one case each. These five cases were excluded from this study. We also excluded 30 cases who underwent LG by junior surgeons.

Finally, 403 patients underwent MIS, including 197 RG and 206 LG surgeries. Among the MIS cases, RG was mainly performed for GC from April 2018 to July 2022 because Japanese National Insurance approved financial support for RG in April 2018. RG and LG surgical procedures at our institution have been previously reported.^15^ Three surgeons performed all RG and LG procedures in this study. All three surgeons have experienced more than 50 LG cases and are certified by the Japanese Endoscopic Surgical Society (JESA), and also possess RG certificates issued by an Intuitive Corporation. Regarding RG types, two surgeons performed Ultrasonic Based Robotic Gastrectomy reported by Hyung et al.^2^ The other surgeon performed Maryland Bipolar‐Based Robotic Gastrectomy, as reported by Uyama et al.^3^

### 2.2 VFA measurement

VFA is determined by measuring the intra‐abdominal fat density area of an axial slice of an abdominal CT image at the height of the patient's umbilicus. VFA is calculated by setting the attenuation level within the range of −200 and −50 threshold unit using the image analyzing system SYNAPSE VINCENT® (FUJI film corporation) reported in a previous study.^11^ To determine the optimal cutoff point of the VFA values, receiver operating characteristic (ROC) curve were generated using the presence of severe intra‐abdominal infectious complications (IAICs) as the endpoint. The maximum Youden index, which was calculated by the ROC curve, was determined to be the optimal cutoff point for VFA. The patients were placed in either the high or low VFA group according to the cutoff point.

### 2.3 Outcomes

We measured surgical time, estimated surgical blood loss, the number of retrieved LNs, postoperative complications, the severity of complications, the rate of readmission and re‐operation, and the duration of postoperative stay.

The Clavien–Dindo (CD) classification was followed to evaluate postoperative complication severity.^16^ We defined overall postoperative complications as Grade II or greater severity as classified by the CD system. Severe complications were those with a severity greater than Grade IIIa. We defined pancreatic fistula, anastomotic leakage, leakage at the duodenal stump, and intra‐abdominal abscess as intra‐abdominal infectious complications (IAICs).

### 2.4 Postoperative management

Prophylactic antibiotics were administered intraoperatively every 3 h. Patients generally started a liquid diet on postoperative day (POD) 1 and a soft diet on POD 3. If infectious complications developed after surgery, broad‐spectrum antibiotics were administered. If intra‐abdominal complications occurred after surgery, such as anastomotic leakage, bowel obstruction, pancreatic fistula, and intra‐abdominal abscess, appropriate interventions (i.e., radiographic or surgical interventions and conservative pharmacological treatment), and parenteral venous nutrition and fasting were carried out according to the severity of those complications. Patients were discharged from the hospital around POD 13 if their condition was stable enough to resume daily life at home. Some patients required longer hospital stays due to reduced food intake and poor home environments; the need for an extended stay was determined on a case‐by‐case basis.

The pathological diagnosis and classifications of GC were made following the JGCA guidelines^17^ and the Union for International Cancer Control TNM Classification of Malignant Tumors (8th edition).^18^ All data were extracted from a prospectively registered database. This study was approved by the Ethics Committee of Osaka City General Hospital (No. 1806031). All patients provided informed consent.

### 2.5 Propensity score matching

We performed 1:1 PS matching to reduce the heterogeneity of patients' backgrounds between the RG and LG groups. A PS was calculated as the conditional probability of receiving cases from either group using a logistic regression model and included age, sex, ASA‐PS score, clinical and pathological oncological stage, BMI, VFA, type of gastrectomy, the extent of lymph node dissection, and surgical experiences of three surgeons.

### 2.6 Statistical analysis

Continuous variables were compared using Mann–Whitney's *U* test, and categorical variables were compared using the chi‐squared test or Fisher's exact test. Differences were considered significant for *p* values <0.05. SPSS and EZR (Saitama Medical Center, Jichii Medical University, Saitama, Japan) software were used for data analysis.

## 3 RESULTS

### 3.1 The ROC curve of VFA

**FIGURE 2:**
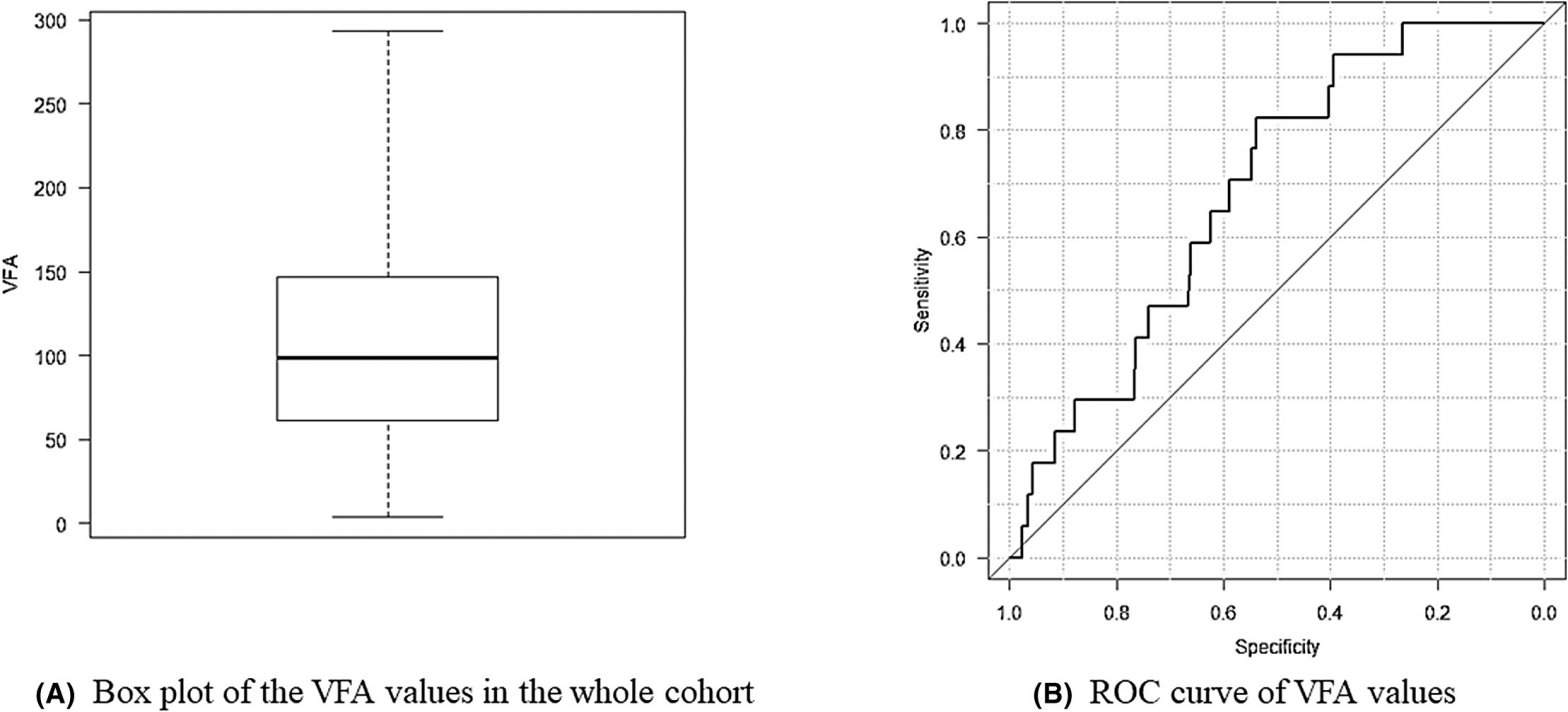
Box plot of the VFA values in the whole cohort (A) and receiver operating characteristic (ROC) curve of VFA modulated by presence/absence of severe IAICs (B). The median of VFA values in this study was 98.2 cm^2^. The area under the curve (AUC) for VFA was 0.681. At a VFA of 106 cm^2^, the Youden index was maximized.

Figure 2 shows the box plot and the ROC curve of VFA modulated by presence/absence of severe IAICs. The median VFA value in the whole cohort was 98.2 cm^2^. Interquartile range (IQR) was 61.4–147. The area under the curve (AUC) of the ROC curve was 0.686. The maximum Youden index for VFA was 106 cm^2^, which was determined to be the VFA cutoff value. All patients were assigned to a “high” (*n* = 188) or “low” (*n* = 215) VFA group based on where that patient's VFA was relative to the threshold of 106 cm^2^.

### 3.2 Association of VFA values with baseline characteristics and surgical outcomes in the whole cohort

The relationships between VFA and patients' characteristics and surgical outcomes are shown in Table 1. The median VFA value in the high and low VFA groups were 153.1 and 60.1 cm^2^, respectively. BMI values in the high and low VFA groups were 24.9 and 21.4, respectively. There were more males, fewer patients classified as ASA‐PS category I, and more patients classified as ASA‐PS category II in the high VFA group compared to the low VFA group. There were no significant differences in the two groups' mean age, oncological stage, type of gastrectomy, and extent of LN dissection. Regarding surgical outcomes, surgical time was significantly longer for high VFA patients (389 vs. 356 min *p* < 0.001), and estimated blood loss during surgery was significantly greater (50 vs. 20 mL, *p* < 0.001). Patients in the high VFA group had significantly fewer retrieved LNs (28 vs. 31, *p* < 0.01). There were significantly more postoperative overall and severe complications among high VFA patients (CD Grade II or higher; 23.1% vs. 9.8% *p* < 0.001; CD Grade III or higher; 10.6% vs. 3.3%, *p* = 0.004). There were significantly more IAICs and more‐severe IAICs in the high VFA group (CD Grade II or higher 12.8% vs. 1.9%, *p* < 0.001; CD Grade III or higher 8.0% vs. 1.4%, *p* < 0.001). Postoperatively, anastomotic leakage, intra‐abdominal abscesses, and pancreatic fistulas were more common in high VFA patients (leakage; 5.9% vs. 0.5%, *p* = 0.002; abscess; 6.2% vs. 1.7%, *p* = 0.024; pancreatic fistula; 3.1% vs. 0.4% *p* = 0.041). Hospital stay after surgery was significantly longer in the high VFA group than in the low VFA group (11.0 vs. 10.0 days, interquartile range (IQR) 10.0–14.0 vs. 9.0–12.0, *p* = 0.001).

**TABLE 1:** Patient characteristics and surgical outcomes stratified by VFA value.

	Low VFA (*n* = 214)	High VFA (*n* = 188)	*p* Value
Age^a^	67.6 (31–91)	68.7 (37–93)	0.369
Gender			<0.001
Male	105 (49.1%)	144 (76.6%)	
Female	109 (50.9%)	44 (23.4%)	
ASA‐PS			0.009
I	44 (20.4%)	18 (9.6%)	
II	143 (67.1%)	144 (76.6%)	
III	26 (12.5%)	26 (13.8%)	
BMI^a^	21.4 (15.3–32.0)	24.9 (17.8–44.2)	<0.01
VFA^a^	60.1 (3.5–105.3)	153.1 (106.1–293.0)	<0.01
cStage			0.104
I	134 (62.6%)	102 (54.3%)	
II/III	80 (37.4%)	86 (45.7%)	
pT			0.520
T1	134 (62.6%)	104 (55.3%)	
T2	22 (10.3%)	22 (11.7%)	
T3	36 (16.8%)	39 (20.7%)	
T4	22 (10.3%)	23 (12.2%)	
pN			0.792
N0	144 (67.6%)	120 (63.8%)	
N1	39 (18.3%)	29 (19.1%)	
N2	15 (7.0%)	13 (7.4%)	
N3	15 (7.0%)	14 (9.6%)	
pStage			0.106
I	135 (63.1%)	103 (54.8%)	
II	40 (18.7%)	51 (27.1%)	
III	39 (18.2%)	33 (17.6%)	
IV	0	1 (0.5%)	
Type of surgical approach			0.046
Robotic	115 (53.7%)	82 (43.6%)	
Laparoscopic	90 (46.3%)	106 (56.4%)	
Type of gastrectomy			0.633
DG	184 (86.0%)	155 (82.4%)	
TG	24 (11.2%)	26 (13.8%)	
PG	6 (2.8%)	7 (3.7%)	
Extent of LN dissection			0.156
D1/D1+	132 (61.7%)	102 (54.3%)	
D2	82 (38.3%)	86 (45.7%)	
Surgical time, min^b^	356 (302–415)	389 (340–457)	<0.001
Surgical blood loss, mL^b^	20 (10–50)	50 (30–100)	<0.001
The number of retrieved lymph nodes^b^	31 (24–41)	28 (20–34.7)	<0.01
Clavien‐Dindo			0.004
Grade 0	186 (86.9%)	135 (71.8%)	
Grade I	6 (2.8%)	11 (5.9%)	
Grade II	15 (7.0%)	22 (11.7%)	
Grade III	5 (2.3%)	15 (8.0%)	
Grade IV	1 (0.5%)	3 (1.6%)	
Grade V	1 (0.5%)	2 (1.1%)	
Overall complication^c^	21 (9.8%)	44 (23.1%)	0.001
Severe complication^d^	7 (3.3%)	20 (10.6%)	0.004
Overall IAICs^c^	4 (1.9%)	24 (12.8%)	0.001
Severe IAICs^d^	3 (1.4%)	15 (8.0%)	0.001
Intraabdominal abscess^c^	3 (1.4%)	11 (5.9%)	0.026
Pancreatic fistula^c^	0	6 (3.2%)	0.010
Anastomotic leakage^c^	1 (0.5%)	11 (5.9%)	0.002
Systemic complication^c^	7 (3.3%)	10 (5.4%)	0.329
Reoperation	3 (1.4%)	5 (2.7%)	0.482
Readmission	4 (1.9%)	5 (2.7%)	0.739
Duration of postoperative stay, days^b^	10.0 (9–12)	11.0 (10–14)	0.001

Abbreviations: ASA‐PS, American Society of Anesthetists‐Physical status; BMI, body mass index; VFA, visceral fat area; DG, distal gastrectomy; IAICs, intraabdominal infectious complications; PG; proximal gastrectomy; TG, total gastrectomy.

^a^ Data were expressed as mean (range).

^b^ Data were expressed as median (interquartile range).

^c^ Clavien–Dindo classification Grade II or higher.

^d^ Clavien–Dindo classification Grade III or higher.

### 3.3 Association of surgical approaches with baseline characteristics and surgical outcomes in the low VFA group

Tables 2 and 3 show the patients' backgrounds and surgical outcomes before and after PS matching for the low VFA group. Before PS matching, there were more patients with more‐advanced oncological stages and low VFA in the RG group. After PS matching, 77 patients' backgrounds were well balanced with similar BMI and VFA values (BMI; 21.0 vs. 20.9, *p* = 0.932, VFA; 65.9 vs. 64.7, *p* = 0.453). Regarding surgical outcomes, surgical time was significantly longer in the RG group than in the LG group (378 vs. 312 min, *p* = 0.001), while estimated surgical blood loss was similar between the two groups (15.0 vs. 20.0 mL, *p* = 0.167). Overall complication rate was significantly higher in the RG group than in the LG group (18.2% vs. 5.2%, *p* = 0.022), while severe complication was equivalent between the two groups (5.2% vs. 2.6%, *p* = 0.681). Overall and severe IAIC were similar between the two groups (CD Grade II or higher IAIC 2.6% vs. 2.6%, *p* = 1.0; CD Grade III or higher IAIC 1.1% vs. 2.6%, *p* = 1.0). There were no significant between‐group differences for readmission and re‐operation rate. Hospital stay after surgery was similar between the two groups (10.0 vs. 10.0 days, *p* = 0.23). The rate of total surgical site complications, including gastric delayed emptying, bowel obstruction, and chyle leakage, were significantly higher in the RG group than in the LG group (15.6% vs. 3.9%, *p* = 0.027), while medical complications were similar between the two groups.

**TABLE 2:** Patient characteristics in the low VFA group.

Characteristic	Whole cohort			Matched cohort		
	Robot (*n* = 115)	Laparoscopic (n = 100)	*p* Value	Robot (*n* = 77)	Laparoscopic (*n* = 77)	*p* Value
Age^a^	69.1 (34–91)	65.9 (31–86)	0.079	67.7 (34–91)	66.8 (31–86)	0.681
Gender			0.683			0.747
Male	55 (47.8%)	51 (51.0%)		41 (52.2%)	38 (49.4%)	
Female	60 (52.2%)	49 (49.0%)		36 (47.8%)	39 (50.6%)	
ASA‐PS			0.567			1.00
I	21 (18.3%)	24 (24.2%)		17 (22.1%)	17 (22.1%)	
II	80 (69.6%)	63 (63.6%)		47 (61.0%)	48 (62.3%)	
III	14 (12.2%)	12 (12.1%)		13 (16.9%)	12 (15.6%)	
BMI^b^	21.0 (19.4–23.3)	21.4 (19.9–23.1)	0.542	21.0 (19.3–23.3)	20.9 (19.0–22.9)	0.932
VFA^b^	57.8 (30.9–84.2)	71.9 (47.4–88.0)	0.015	65.9 (41.5–88.2)	64.7 (36.1–82.3)	0.453
cStage			0.007			1.00
I	62 (53.9%)	72 (72.0%)		51 (66.2%)	51 (66.2%)	
II/III	53 (46.1%)	28 (28.0%)		26 (33.8%)	26 (33.8%)	
pT			0.201			0.732
T1	70 (60.9%)	64 (64.0%)		60 (65.2%)	52 (56.5%)	
T2	8 (7.0%)	14 (14.0%)		5 (5.4%)	11 (12.0%)	
T3	23 (20.0%)	14 (14.0%)		18 (19.6%)	19 (20.7%)	
T4	14 (12.2%)	8 (8.0%)		9 (9.8%)	10 (10.9%)	
pN			0.632			0.474
N0	74 (64.3%)	70 (70.0%)		62 (67.4%)	57 (62.6%)	
N1	21 (18.3%)	18 (18.0%)		14 (15.2%)	22 (24.2%)	
N2	10 (8.7%)	6 (6.0%)		8 (8.7%)	6 (6.6%)	
N3	10 (8.7%)	6 (6.0%)		8 (8.7%)	6 (6.6%)	
pStage			0.036			0.862
I	63 (54.8%)	72 (72.0%)		51 (66.2%)	51 (59.8%)	
II	26 (22.6%)	14 (14.0%)		16 (20.8%)	14 (21.7%)	
III	26 (22.6%)	14 (14.0%)		10 (13.0%)	12 (17.4%)	
Type of gastrectomy			0.388			1.00
DG	96 (83.5%)	89 (89.0%)		69 (89.6%)	70 (90.9%)	
TG	16 (13.9%)	8 (8.0%)		7 (9.1%)	7 (9.1%)	
PG	3 (2.6%)	3 (3.0%)		1 (1.3%)	0	
Extent of LN dissection			0.676			0.620
D1/D1+	69 (60.0%)	63 (63.0%)		49 (63.6%)	45 (58.4%)	
D2	46 (40.0%)	37 (37.0%)		28 (36.4%)	32 (41.6%)	
Patient number who underwent surgery by three surgeons			0.812			0.912
Surgeon A	38 (33.0%)	33 (33.0%)		25 (32.5%)	25 (32.5%)	
Surgeon B	38 (33.0%)	31 (31.0%)		25 (32.5%)	25 (32.5%)	
Surgeon C	39 (34.0%)	36 (36.0%)		27 (35.0%)	27 (35.0%)	

Abbreviations: ASA‐PS, American Society of Anesthetists‐Physical status; BMI, body mass index; DG, distal gastrectomy; PG, proximal gastrectomy; TG, total gastrectomy.

^a^ Data were expressed as mean (range).

^b^ Data were expressed as median (interquartile range).

**TABLE 3:** Surgical outcomes in patients with the low VFA.

	Whole cohort			Matched cohort		
	Robot (*n* = 115)	Laparoscopic (*n* = 100)	*p* Value	Robot (*n* = 77)	Laparoscopic (*n* = 77)	*p* Value
Surgical time, min^a^	386.0 (343–440)	312.0 (275–364)	0.001	378 (346–418)	312 (274–370)	0.001
Surgical blood loss, mL^a^	20.0 (10–40)	25.0 (10–50)	0.180	15.0 (7–40)	20.0 (10–50)	0.167
The number of retrieved lymph nodes^a^	29 (23–39)	32 (24–42)	0.236	29 (24–39)	32 (26–44)	0.387
Clavien‐Dindo			0.209			0.023
Grade 0	95 (82.6%)	92 (92.0%)		59 (76.6%)	71 (92.2%)	
Grade I	4 (3.5%)	2 (2.0%)		3 (3.9%)	2 (2.6%)	
Grade II	11 (9.6%)	4 (4.0%)		11 (14.3%)	2 (2.6%)	
Grade III	4 (3.5%)	1 (1.0%)		3 (3.9%)	1 (1.3%)	
Grade IV	0	1 (1.0%)		0	1 (1.3%)	
Grade V	1 (0.9%)	0		1 (1.3%)	0	
Overall complication^b^	15 (13.0%)	6 (6.0%)	0.107	14 (18.2%)	4 (5.2%)	0.022
Severe complication^c^	5 (4.3%)	2 (2.0%)	0.454	4 (5.2%)	2 (2.6%)	0.681
Overall IAICs^b^	2 (1.7%)	2 (2.0%)	1.00	2 (2.6%)	2 (2.6%)	1.00
Severe IAICs^c^	1 (0.9%)	2 (2.0%)	0.599	1 (1.1%)	2 (2.6%)	1.00
Surgical site complications^b^	15 (13.0%)	5 (5.0%)	0.058	12 (15.6%)	3 (3.9%)	0.027
Intra‐abdominal abscess	2	1		2	1	
Anastomotic leakage	0	1		0	1	
Pancreatic fistula	0	0		0	0	
Bleeding	1	0		1	0	
Bowel obstruction	3	0		2	0	
Gastric delayed emptying	4	1		3	1	
Chyle leakage	2	1		2	0	
NOMI	1	0		1	0	
Superficial SSI	2	1		1	0	
Medical complications^b^	5 (4.3%)	2 (2.0%)	0.455	5 (6.5%)	2 (2.6%)	0.442
Reoperation	3 (2.6%)	0	0.210	2 (2.2%)	0	0.497
Readmission	4 (3.5%)	0	0.125	4 (4.3%)	0	0.121
Duration of postoperative stay, days^a^	10.0 (9.0–13.0)	10.0 (9.0–12.0)	0.451	10.0 (9.0–13.0)	10.0 (9.0–12.0)	0.651

Abbreviations: IAICs; intraabdominal infectious complication, NOMI; non‐occlusive mesentery ischemia, SSI; surgical site infection.

^a^ Data were expressed as median (interquartile range).

^b^ Clavien–Dindo classification Grade II or higher.

^c^ Clavien–Dindo classification Grade III or higher.

### 3.4 Association of surgical approaches with baseline characteristics and surgical outcomes in the high VFA group

Tables 4 and 5 show the patients' backgrounds and surgical outcomes before and after PS matching in the high VFA group. Before PS matching, the RG group had significantly more females than the LG group. After PS matching, each of the 71 patients in the RG group and LG group were selected. The groups were considered balanced for patient sex. The median BMI and VFA values in the RG and LG groups were similar after PS matching (BMI; 24.3 vs. 24.6, *p* = 0.69, VFA; 148 vs. 144, *p* = 0.85). The RG group had a significantly longer median operation time; however, the median surgical blood loss was similar between the two groups (operation time; 417 vs. 376 min *p* < 0.001; surgical blood loss; 50 vs. 50 mL, *p* = 0.62). Patients in the RG group have significant fewer severe complications (CD Grade III or higher; 4.2% vs. 16.9%, *p* = 0.026). In addition, the severe IAIC rate was significantly lower in the RG group than in the LG group (CD Grade III or higher IAIC; 1.4% vs. 15.4%, *p* < 0.004). Hospital stay after surgery was significantly shorter in RG group than in LG group (11.0 vs. 12.0 days, *p* = 0.039). Regarding individual post‐surgical morbidities, there were no significant between‐group differences (Table 6).

**TABLE 4:** Patient characteristics in the high VFA group.

Characteristic	Whole cohort			Matched cohort		
	Robot (*n* = 82)	Laparoscopic (*n* = 106)	*p* Value	Robot (*n* = 71)	Laparoscopic (*n* = 71)	*p* Value
Age^a^	68.3 (37–93)	69.1 (42–89)	0.594	68.2 (37–93)	68.1 (42–84)	0.964
Gender			0.056			1.00
Male	57 (69.5%)	87 (82.1%)		55 (77.5%)	56 (78.9%)	
Female	25 (30.5%)	19 (17.9%)		16 (22.5%)	15 (21.1%)	
ASA‐PS			0.242			0.357
I	10 (12.2%)	8 (7.5%)		10 (14.1%)	4 (8.5%)	
II	58 (70.7%)	86 (81.1%)		51 (71.8%)	45 (83.1%)	
III	4 (17.1%)	12 (11.3%)		10 (14.1%)	5 (8.5%)	
BMI^b^	24.3 (18.6–37.2)	24.5 (18.6–44.2)	0.618	24.3 (20–37)	24.6 (18–42)	0.691
VFA^b^	147.7 (127–169)	149.1 (122–174)	0.952	148 (113–249)	144 (112–293)	0.851
cStage			1.00			0.501
I	44 (53.7%)	58 (54.7%)		41 (57.7%)	36 (50.7%)	
II/III	38 (46.8%)	48 (45.3%)		30 (42.3%)	35 (49.3%)	
pT			0.797			0.237
T1	47 (59.2%)	57 (53.8%)		43 (60.6%)	36 (50.7%)	
T2	11 (13.2%)	11 (10.4%)		10 (14.1%)	6 (8.5%)	
T3	15 (17.1%)	24 (22.6%)		11 (15.5%)	19 (26.8%)	
T4	9 (10.5%)	14 (13.2%)		7 (9.9%)	10 (14.1%)	
pN			0.126			0.507
N0	51 (62.2%)	69 (65.1%)		47 (66.2%)	42 (59.2%)	
N1	12 (14.6%)	24 (22.6%)		10 (14.1%)	17 (23.9%)	
N2	7 (8.5%)	7 (6.6%)		6 (8.5%)	6 (8.5%)	
N3	12 (14.6%)	6 (5.7%)		8 (11.3%)	6 (8.5%)	
pStage			0.154			0.654
I	45 (54.9%)	58 (54.7%)		41 (57.7%)	36 (50.7%)	
II	18 (22.0%)	33 (31.1%)		17 (23.9%)	21 (29.6%)	
III	19 (23.2%)	14 (13.2%)		13 (18.3%)	14 (19.7%)	
IV	0	1 (0.9%)		0	0	
Type of gastrectomy			0.063			1.00
DG	67 (81.7%)	88 (83.0%)		61 (85.9%)	61 (85.9%)	
TG	9 (11.0%)	17 (16.1%)		9 (12.7%)	9 (12.7%)	
PG	6 (7.3%)	1 (0.9%)		1 (1.4%)	1 (1.4%)	
Extent of LN dissection			0.661			0.865
D1/D1+	46 (56.1%)	56 (52.8%)		40 (56.3%)	42 (59.2%)	
D2	36 (43.9%)	50 (47.2%)		31 (43.7%)	29 (40.8%)	
Patient number who underwent surgery by three surgeons			0.890			0.731
Surgeon A	27 (32.9%)	35 (33.0%)		25 (35.2%)	24 (33.8%)	
Surgeon B	29 (35.4%)	36 (34.0%)		25 (35.2%)	25 (35.2%)	
Surgeon C	26 (31.7%)	35 (33.0%)		21 (29.6%)	22 (31.0%)	

^a^ Data were expressed as mean (range).

^b^ Data were expressed as median (interquartile range).

**TABLE 5:** Surgical outcomes in patients with the high VFA.

	Whole cohort			Matched cohort		
	Robot (*n* = 82)	Laparoscopic (*n* = 106)	*p* Value	Robot (*n* = 71)	Laparoscopic (*n* = 71)	*p* Value
Surgical time, min^a^	420 (356–483)	371 (323–424)	0.012	417 (350–482)	376 (321–436)	0.015
Surgical blood loss, mL^a^	50 (20–87)	50 (30–100)	0.315	50 (30–85)	50 (30–100)	0.621
The mean number of retrieved lymph nodes	29.0 (19–36)	28.0 (20–34)	0.725	28.0 (19–35)	28.0 (19–34)	0.799
Clavien–Dindo			0.169			0.030
Grade 0	61 (74.4%)	74 (69.8%)		54 (76.1%)	52 (73.2%)	
Grade I	7 (8.5%)	4 (3.8%)		7 (9.9%)	1 (1.4%)	
Grade II	9 (11.0%)	13 (12.3%)		7 (9.9%)	6 (8.5%)	
Grade III	3 (2.7%)	12 (11.3%)		2 (2.8%)	9 (12.7%)	
Grade IV	2 (2.4%)	1 (0.9%)		1 (1.4%)	1 (1.4%)	
Grade V	0	2 (1.9%)		0	2 (2.8%)	
Overall complication^b^	16 (19.5%)	28 (26.4%)	0.301	12 (16.9%)	17 (23.9%)	0.405
Severe complication^c^	5 (6.1%)	15 (14.2%)	0.096	3 (4.2%)	12 (16.9%)	0.026
Overall IAICs^b^	8 (9.8%)	16 (15.1%)	0.379	5 (7.0%)	13 (18.3%)	0.075
Severe IAICs^c^	3 (3.7%)	12 (11.3%)	0.062	1 (1.4%)	11 (15.4%)	0.004
Reoperation	2 (2.4%)	3 (2.8%)	1.00	1 (1.4%)	2 (2.8%)	1.00
Readmission	3 (3.7%)	2 (1.9%)	0.655	3 (4.2%)	2 (2.8%)	1.00
Duration of postoperative stay, days^a^	11.0 (11.0–13.0)	12.0 (10.0–14.7)	0.031	11.0 (11.0–13.0)	12.0 (10.0–14.0)	0.039

Abbreviation: IAIC, intraabdominal infectious complication.

^a^ Data were expressed as median (interquartile range).

^b^ Clavien–Dindo classification Grade II or higher.

^c^ Clavien–Dindo classification Grade III or higher.

**TABLE 6:** Grading of postoperative complications according to Clavien–Dindo classification in the high VFA group.

	Whole cohort			Matched cohort		
	Robot (*n* = 82)	Laparoscopic (*n* = 106)	*p* Value	Robot (*n* = 71)	Laparoscopic (*n* = 71)	*p* Value
Anastomotic leakage						
Duodenal stump leakage						
Grade 0	78	99		69	65	
Grade I	0	0		0	0	
Grade II	1	0		1	0	
Grade IIIa	1	5		0	4	
Grade IIIb	1	0		1	0	
Grade IV	1	1		0	1	
Grade V	0	1		0	1	
Grade II or higher	4 (4.9%)	7 (6.6%)	0.759	2 (2.8%)	6 (8.5%)	0.275
Grade III or higher	3 (3.7%)	7 (6.6%)	0.517	1 (1.4%)	6 (8.5%)	0.116
Pancreatic fistula						
Grade 0	81	101		70	66	
Grade I	0	0		0	0	
Grade II	1	1		1	1	
Grade IIIa	0	3		0	3	
Grade IIIb	0	0		0	0	
Grade IV	0	0		0	0	
Grade V	0	1		0	1	
Grade II or higher	1 (1.2%)	5 (4.7%)	0.234	1 (1.4%)	5 (7.0%)	0.209
Grade III or higher	0	4 (3.8%)	0.133	0	4 (5.6%)	0.120
Intra‐abdominal abscess						
Grade 0	79	98		53	50	
Grade I	0	0		0	0	
Grade II	3	3		2	1	
Grade IIIa	0	5		0	4	
Grade IIIb	0	0		0	0	
Grade IV	0	0		0	0	
Grade V	0	0		0	0	
Grade II or higher	3 (3.7%)	8 (7.5%)	0.353	2 (2.8%)	5 (7.0%)	0.411
Grade III or higher	0	5 (4.7%)	0.069	0	4 (5.6%)	0.120
Superficial SSI	1 (1.1%)	1 (0.9%)	1.00	1	0	1.00
Grade II	1	1		1 (1.4%)	0	
Necrosis of remnant stomach	0	1 (0.9%)	1.00	0	1 (1.4%)	1.00
Grade V		1		0	1	
Bowel obstruction	3 (3.7%)	3 (2.8%)	1.00	2 (2.8%)	1 (1.4%)	1.00
Grade I	1	0		0	0	
Grade II	1	1		1	1	
Grade IIIa	1	1		1	0	
Grade IIIb	0	1		0	0	
Gastric delayed emptying	3 (3.7%)	2 (1.9%)	0.655	2 (2.8%)	1 (1.4%)	1.00
Grade II	3	2		2	1	
Chyle leakage	1 (1.1%)	0	1.00	1 (1.4%)	0	1.00
Grade I	1			1		
Surgical site complication			0.849			0.516
Grade II or higher	14 (17.1%)	20 (18.9%)		11 (15.5%)	15 (21.1%)	
Pneumonia	4 (4.9%)	5 (4.7%)	1.00	3 (4.2%)	1 (2.8%)	1.00
Grade II	3	5		3	1	
Grade IV	1					
Septic shock	2 (2.4%)	1 (0.9%)	0.581	1 (1.4%)	0	1.00
Grade II	2	1		1	0	
Cervical infarction	0	1 (0.9%)	1.00	0	0	1.00
Grade II		1				
Medical complications			1.00			1.00
Grade II or higher	4 (5.1%)	6 (5.7%)		3 (4.2%)	3 (4.2%)	

## 4 DISCUSSION

We found that patients with high VFA and GC had longer operation times, more bleeding, and more postoperative complications such as anastomotic leakage, intra‐abdominal abscesses, and pancreatic fistulas than patients with low VFA. These results have been previously reported.^11^, ^19^, ^20^ Generally, increased abdominal cavity fat makes surgery difficult due to the increased likelihood of bleeding, difficulty in recognizing the appropriate dissection layer around the pancreas, and a smaller abdominal cavity surgical field. Laparoscopic gastrectomy for patients with visceral obesity is technically demanding because articulation surgical devices are not used. We investigated the impact of a robotic system on postoperative complication rates in patients with visceral obesity. We found that the robotic system was associated with fewer severe IAIC in patients with visceral obesity. Robotic systems have many advantages, such as an articulated endo‐wrist, tremor filtering, and a superior surgical view. These advantages enable us to perform safe and meticulous lymph node dissection around the pancreas, which may result in less severe IAICs in patients with visceral obesity.

Although there have been previous reports on the effects of RG on surgical outcomes in obese patients, few studies have shown the short‐term outcome benefits.^21^, ^22^ Previous studies have used the BMI as a surrogate marker for obesity and investigated the efficacy of BMI in predicting surgical outcomes. Some reports have shown that VFA is a better surrogate obesity marker for IAIC than BMI.^12^, ^23^ VFA precisely reflects the amount of intra‐abdominal visceral fatty tissue, whereas BMI reflects the muscle and fatty tissue in the whole body. Moreover, females, in particular, can sometimes have a high BMI and normal VFA since females tend to deposit fat subcutaneously and not in the abdominal cavity.^24^ Therefore, we used VFA as a surrogate obesity marker for IAIC after surgery. To the best of our knowledge, only two studies have examined the impact of RG on surgical outcomes in patients with a high VFA. These two studies yielded different results. One report by Hikage et al. showed that pancreatic fistulas were less common in patients with high VFA in the RG group than in the LG group.^25^ Another report showed that the surgical outcomes following distal gastrectomy in patients with visceral obesity were similar between the RG and LG groups.^26^ Our study showed that severe IAICs were significantly less common in RG than in LG. However, we could not detect the exact type of complications among the IAICs. In our study, patients with a high VFA in the RG group tended to have less severe anastomotic leakage, pancreatic fistulas, and intra‐abdominal abscesses. However, these results were not statistically significant (anastomotic leakage, 1.4% vs. 8.5%, *p* = 0.116; pancreatic fistula, 0% vs. 5.6%, *p* = 0.120; and abscess, 0% vs. 5.6%, *p* = 0.120). Large‐scale prospective multicenter trials and national databases are required to clarify the exact types of complications experienced by these patients. By contrast, in the low VFA group, the postoperative IAIC rates were similar between the RG and LG groups. However, the total number of surgical site complications after PS matching was significantly higher in the RG group than in the LG group. Surgical site complications in the RG in the low VFA group included bleeding in one case, bowel obstruction in two cases, delayed gastric emptying in three cases, and chyle leakage in two cases. We could not provide an exact explanation for more surgical site complications in the RG than in the LG in the low VFA group. Both patients in the RG who developed bowel obstruction had a history of open colon cancer surgery. Moreover, all three patients who developed delayed gastric emptying underwent Billroth I reconstruction (gastroduodenostomy) after gastrectomy and had a history of open cholecystectomy. Ojima et al.^27^ reported that a history of abdominal surgery was an independent risk factor for postoperative complications after robotic gastrectomy for gastric cancer. The history of previous abdominal surgery might be associated with a higher incidence of surgical site complications in RG patients in the low VFA group. In this study, the surgical time was significantly longer in the RG group than in the LG group. Many studies have reported longer operation times and higher RG costs than LG.^9^, ^28^ According to these reports and ours, RG may not provide sufficient short‐term surgical benefits in patients with low VFA and GC.

There have been certain reports on the impact of Ultrasonic Shears (US) or bipolar devices as the main energy device for pancreatic thermal injury. Suda et al. reported that pancreatic fistulas after GC surgeries were significantly less common in RG cases using a bipolar device than in LG cases using a US as the main energy devise.^7^ They speculated that thermal damage to the pancreatic parenchyma was likely to be less in the bipolar device than in the US. Recently, a randomized controlled study comparing the short‐term surgical results of laparoscopic gastrectomy for GC between the groups using US and those using bipolar devices was published in Korea.^29^ The results showed that postoperative CRP levels were significantly higher in the US group than in the bipolar group. The authors speculated that the higher CRP levels in the US group might be associated with pancreatic thermal damage. In this study, all surgeons in the LG group used a US as the main energy device, whereas in the RG group, two surgeons used a US and one surgeon used a bipolar device as the main energy device. However, in the low VFA group, no pancreatic fistulas were observed in the RG and LG groups, whereas in the high VFA group, pancreatic fistulas were observed in one (1.2%) and five (4.7%) cases in the RG and LG groups, respectively. Moreover, in our previous study on the impact of the main energy devices (US and bipolar) used in the RG on short‐term surgical results after GC surgery, no correlation was observed between the surgical devices and pancreatic fistula (0% (0/113) in the US group versus 1.7% (1/58) in the bipolar group; *p* = 0.339).^30^ These results suggested that pancreatic fistula was not associated with the type of energy device but with visceral obesity.

### 4.1 Limitations

First, this was a retrospective, single institution study. Second, we could not precisely quantify the surgeons' RG and LG skills; this could have created between‐group differences in short‐term outcomes. All of three surgeons in this study qualified endoscopic surgical skill qualification system of the Japanese society of endoscopic surgery and experienced LG and RG equally. Therefore, the surgeons were expected to have comparable surgical skills. Moreover, we used the numbers of patients who underwent RG and LG performed by each surgeon as a covariate in PS matching analysis to reduce the surgeon's technical bias. Lastly, only short‐term outcomes after RG and LG were investigated. Thus, the impact of the robotic system on long‐term outcomes requires further study.

## 5 CONCLUSION

RG could be an alternative to LG because of the reduced postoperative IAIC in patients with visceral obesity and GC; however, RG may not benefit non‐viscerally obese patients.

## AUTHOR CONTRIBUTIONS

Study Design: NK. Data collection: NK, KS, TH, YT, YI, TN, SS, and TI. Statistical analysis and interpretation of results: NK. Drafting of the manuscript: NK. Supervision: KM and YN.

## CONFLICT OF INTEREST STATEMENT

The authors declare no conflicts of interest for this article.

## FUNDING INFORMATION

This study did not receive support from any organization.

## ETHICS STATEMENT

Approval of the research protocol: This study was conducted in accordance with the ethical principles of the Declaration of Helsinki. This was a retrospective study approved by the review board of Osaka City General Hospital.

Informed consent: Patients provided written informed consent.

Registry and the Registration No. of the study/trial: N/A.

Animal Studies: N/A.

## References

[1] Bray F , Ferlay J , Soerjomataram I , Siegel RL , Torre LA , Jemal A . Global cancer statistics 2018: GLOBOCAN estimates of incidence and mortality worldwide for 36 cancers in 185 countries. CA Cancer J Clin. 2018;68(6):394–424. 10.3322/caac.21492 30207593

[2] Hyung WJ , Woo Y , Noh SH . Robotic surgery for gastric cancer: a technical review. J Robot Surg. 2011;5(4):241–249. 10.1007/s11701-011-0263-x 27628113

[3] Uyama I , Kanaya S , Ishida Y , Inaba K , Suda K , Satoh S . Novel integrated robotic approach for suprapancreatic D2 nodal dissection for treating gastric cancer: technique and initial experience. World J Surg. 2012;36(2):331–337. 10.1007/s00268-011-1352-8 22131088

[4] Dalsgaard T , Jensen MD , Hartwell D , Mosgaard BJ , Jorgensen A , Jensen BR . Robotic surgery is less physically demanding than laparoscopic surgery: paired cross sectional study. Ann Surg. 2020;271(1):106–113. 10.1097/SLA.0000000000002845 29923873

[5] Uyama I , Suda K , Nakauchi M , Kinoshita T , Noshiro H , Takiguchi S , et al. Clinical advantages of robotic gastrectomy for clinical stage I/II gastric cancer: a multi‐institutional prospective single‐arm study. Gastric Cancer. 2019;22(2):377–385. 10.1007/s10120-018-00906-8 30506394

[6] Ojima T , Nakamura M , Hayata K , Kitadani J , Katsuda M , Takeuchi A , et al. Short‐term outcomes of robotic gastrectomy vs laparoscopic gastrectomy for patients with gastric Cancer: A randomized clinical trial. JAMA Surg. 2021;156(10):954–963. 10.1001/jamasurg.2021.3182 34468701 PMC8411361

[7] Suda K , Man IM , Ishida Y , Kawamura Y , Satoh S , Uyama I . Potential advantages of robotic radical gastrectomy for gastric adenocarcinoma in comparison with conventional laparoscopic approach: a single institutional retrospective comparative cohort study. Surg Endosc. 2015;29(3):673–685. 10.1007/s00464-014-3718-0 25030478

[8] Lu J , Zheng CH , Xu BB , Xie JW , Wang JB , Lin JX , et al. Assessment of robotic versus laparoscopic distal gastrectomy for gastric Cancer: A randomized controlled trial. Ann Surg. 2021;273(5):858–867. 10.1097/SLA.0000000000004466 32889876

[9] Kim HI , Han SU , Yang HK , Kim YW , Lee HJ , Ryu KW , et al. Multicenter prospective comparative study of robotic versus laparoscopic gastrectomy for gastric adenocarcinoma. Ann Surg. 2016;263(1):103–109. 10.1097/SLA.0000000000001249 26020107

[10] Suda K , Yamamoto H , Nishigori T , Obama K , Yoda Y , Hikage M , et al. Safe implementation of robotic gastrectomy for gastric cancer under the requirements for universal health insurance coverage: a retrospective cohort study using a nationwide registry database in Japan. Gastric Cancer. 2022;25(2):438–449. 10.1007/s10120-021-01257-7 34637042 PMC8505217

[11] Tokunaga M , Hiki N , Fukunaga T , Ogura T , Miyata S , Yamaguchi T . Effect of individual fat areas on early surgical outcomes after open gastrectomy for gastric cancer. Br J Surg. 2009;96(5):496–500. 10.1002/bjs.6586 19358176

[12] Yoshikawa K , Shimada M , Kurita N , Iwata T , Nishioka M , Morimoto S , et al. Visceral fat area is superior to body mass index as a predictive factor for risk with laparoscopy‐assisted gastrectomy for gastric cancer. Surg Endosc. 2011;25(12):3825–3830. 10.1007/s00464-011-1798-7 21688079

[13] Collaborators GBDEMRO . Burden of obesity in the eastern Mediterranean region: findings from the global burden of disease 2015 study. Int J Public Health. 2018;63(Suppl 1):165–176. 10.1007/s00038-017-1002-5 PMC5973977 28776243

[14] Collaborators GBDO , Afshin A , Forouzanfar MH , Reitsma MB , Sur P , Estep K , et al. Health effects of overweight and obesity in 195 countries over 25 years. N Engl J Med. 2017;377(1):13–27. 10.1056/NEJMoa1614362 28604169 PMC5477817

[15] Kubo N , Sakurai K , Tamamori Y , Fukui Y , Kuroda K , Aomatsu N , et al. Less severe intra‐abdominal infections in robotic surgery for gastric Cancer compared with conventional laparoscopic surgery: A propensity score‐matched analysis. Ann Surg Oncol. 2022;29(6):3922–3933. 10.1245/s10434-022-11410-w 35181811

[16] Dindo D , Demartines N , Clavien PA . Classification of surgical complications: a new proposal with evaluation in a cohort of 6336 patients and results of a survey. Ann Surg. 2004;240(2):205–213. 10.1097/01.sla.0000133083.54934.ae 15273542 PMC1360123

[17] Japanese Gastric Cancer Association . Japanese gastric cancer treatment guidelines 2014 (ver. 4). Gastric Cancer. 2017;20(1):1–19. 10.1007/s10120-016-0622-4 PMC5215069 27342689

[18] O'Sullivan B , Brierley J , Byrd D , Bosman F , Kehoe S , Kossary C , et al. The TNM classification of malignant tumours‐towards common understanding and reasonable expectations. Lancet Oncol. 2017;18(7):849–851. 10.1016/S1470-2045(17)30438-2 28677562 PMC5851445

[19] Kim MG , Yook JH , Kim KC , Kim TH , Kim HS , Kim BS , et al. Influence of obesity on early surgical outcomes of laparoscopic‐assisted gastrectomy in gastric cancer. Surg Laparosc Endosc Percutan Tech. 2011;21(3):151–154. 10.1097/SLE.0b013e318219a57d 21654297

[20] Wu XS , Wu WG , Li ML , Yang JH , Ding QC , Zhang L , et al. Impact of being overweight on the surgical outcomes of patients with gastric cancer: a meta‐analysis. World J Gastroenterol. 2013;19(28):4596–4606. 10.3748/wjg.v19.i27.4596 23901238 PMC3725387

[21] Park JM , Kim HI , Han SU , Yang HK , Kim YW , Lee HJ , et al. Who may benefit from robotic gastrectomy?: A subgroup analysis of multicenter prospective comparative study data on robotic versus laparoscopic gastrectomy. Eur J Surg Oncol. 2016;42(12):1944–1949. 10.1016/j.ejso.2016.07.012 27514719

[22] Choi S , Song JH , Lee S , Cho M , Kim YM , Hyung WJ , et al. Surgical merits of open, laparoscopic, and robotic gastrectomy techniques with D2 lymphadenectomy in obese patients with gastric Cancer. Ann Surg Oncol. 2021;28(12):7051–7060. 10.1245/s10434-021-09952-6 33834323

[23] Imai Y , Lee SW , Kawai M , Tashiro K , Kawashima S , Tanaka R , et al. Visceral fat area is a better indicator of surgical outcomes after laparoscopic gastrectomy for cancer than the body mass index: a propensity score‐matched analysis. Surg Endosc. 2022;36(5):3285–3297. 10.1007/s00464-021-08642-4 34382123

[24] Xu F , Earp JE , Adami A , Lofgren IE , Delmonico MJ , Greene GW , et al. The sex and race/ethnicity‐specific relationships of abdominal fat distribution and anthropometric indices in US adults. Int J Environ Res Public Health. 2022;19(23):15521. 10.3390/ijerph192315521 36497594 PMC9736224

[25] Hikage M , Fujiya K , Waki Y , Kamiya S , Tanizawa Y , Bando E , et al. Advantages of a robotic approach compared with laparoscopy gastrectomy for patients with high visceral fat area. Surg Endosc. 2022;36(8):6181–6193. 10.1007/s00464-022-09178-x 35294634

[26] Park JY , Ryu KW , Reim D , Eom BW , Yoon HM , Rho JY , et al. Robot‐assisted gastrectomy for early gastric cancer: is it beneficial in viscerally obese patients compared to laparoscopic gastrectomy? World J Surg. 2015;39(7):1789–1797. 10.1007/s00268-015-2998-4 25670040

[27] Ojima T , Hayata K , Kitadani J , Takeuchi A , Yamaue H . Risk factors of postoperative intra‐abdominal infectious complications after robotic gastrectomy for gastric cancer. Oncology. 2022;100(11):583–590. 10.1159/000526920 36273443

[28] Obama K , Sakai Y . Current status of robotic gastrectomy for gastric cancer. Surg Today. 2016;46(5):528–534. 10.1007/s00595-015-1190-7 26019020

[29] Park JH , Kong SH , Berlth F , Choi JH , Kim S , Kim SH , et al. Comparison of perioperative outcomes between bipolar sealing, ultrasonic shears and a hybrid device during laparoscopic gastrectomy for early gastric cancer: a prospective, multicenter, randomized study. Gastric Cancer. 2023;26(3):438–450. 10.1007/s10120-023-01365-6 36735157

[30] Kuroda K , Kubo N , Sakurai K , Tamamori Y , Hasegawa T , Yonemitsu K , et al. Comparison of short‐term surgical outcomes of two types of robotic gastrectomy for gastric Cancer: ultrasonic shears method versus the Maryland bipolar forceps method. J Gastrointest Surg. 2023;27(2):222–232. 10.1007/s11605-022-05527-2 36376726

